# Resveratrol Induces Expression of Metabolic and Antioxidant Machinery and Protects Tilapia under Cold Stress

**DOI:** 10.3390/ijms21093338

**Published:** 2020-05-08

**Authors:** Min-Chen Wang, Yu-Chun Wang, Hui-Wen Peng, Jinn-Rong Hseu, Guan-Chung Wu, Ching-Fong Chang, Yung-Che Tseng

**Affiliations:** 1 Marine Research Station, Institute of Cellular and Organism Biology, Academia Sinica, Taipei 262, Taiwan; mcwinlab@gmail.com (M.-C.W.); b5pickfa@gmail.com (H.-W.P.); 2 Taiwan International Graduate Program, National Taiwan Normal University, Taipei 106, Taiwan; 3 Planning and Information Division, Fisheries Research Institute, COA, Keelung 20246, Taiwan; ycwang@mail.tfrin.gov.tw (Y.-C.W.); jrhseu@mail.tfrin.gov.tw (J.-R.H.); 4 Department of Aquaculture, National Taiwan Ocean University, Keelung 20224, Taiwan; gcwu@mail.ntou.edu.tw (G.-C.W.); b0044@email.ntou.edu.tw (C.-F.C.)

**Keywords:** resveratrol, metabolic adjustment, sirtuin, antioxidation, low temperature, tilapia

## Abstract

Exposures to low ambient temperature require ectothermic fish to not only adjust their metabolic machinery but also to mount protective responses against oxidative stress. In this study, we tested whether diets supplemented with resveratrol (RSV), a naturally occurring polyphenol known to stimulate metabolic and protective responses in various animals, would be beneficial to tilapia (*Oreochromis mossambicus*) under hypothermic challenge. Feeding tilapia with RSV-supplemented diet promoted liver expression of sirtuins and their known targets, including metabolic/antioxidative enzymes. After exposure to 15 °C cold conditions for three days, the oxygen–nitrogen (O:N) ratio was decreased in the control-diet-fed tilapia but not in their RSV-fed counterparts. Moreover, at 27 °C, RSV-fed tilapia showed significantly higher prolonged swim speed compared with controls. RSV feeding produced no significant effect on upper and bottom layer preference between the control- and RSV-treated tilapia at either 27 °C or 15 °C. Together, these findings suggest that RSV stimulates beneficial metabolic/antioxidative adjustments in teleosts and may serve as a valuable feed supplement for tropical fish exposed to cold stress during winter.

## 1. Introduction

Ambient temperature is among the most critical abiotic determinants of animal distribution, behavior and physiological responses [1,2,3,4]. At the molecular level, low temperatures decrease enzymatic reaction rates, while reducing diffusion and transport of biomolecules, and slowing protein denaturation and disaggregation. Low temperatures also inhibit transcription and translation, disrupt cellular cytoskeletal elements, change membrane permeability and affect energy production in cells [5]. In warm-climate-adapted ectotherms, such as tropical fish, low ambient temperatures have been shown to cause rapid reductions in body temperature and are highly associated with poor health and survival [6]. Thus, a physiological cascade of acclimation strategies must be activated in fish species to maintain cellular homeostasis during temperature stress [7,8]. In hypothermia studies, most fish were found to decrease their routine metabolic output in low temperature conditions, which negatively impacts swimming performance; however, this metabolic adjustment seems to allow the organism to sustain a minimum necessary level of aerobic energy production to survive in hypothermal stress. As such, the energy supply is primarily allocated to support maintenance of basic physiological functions [3,9].

Since energy supply is a limiting factor for nearly all physiological processes, fish exposed to cold water must initiate a range of homeostatic responses to offset the passive negative effects of temperature reduction. Therefore, many fish that reside in environments with fluctuating temperatures have developed sophisticated regulatory mechanisms to maintain body core temperature, allowing for continued function of cellular and physiological processes in energy-consuming vital organs and ultimately promoting survival [8]. For example, fish in temperate climates and the Antarctic may convert their energy storage/utilization preference and modify lipids to maintain fluidity of cell membranes and reduce energy consumption when the ambient temperature drops [4,10]. In zebrafish, the utilization of lactate to generate energy equivalents under hypothermic conditions has been proposed to serve as an essential adaptive mechanism supporting cellular and organismic functionality during stress [8,11]. These studies suggested that, despite reductions in the overall metabolic rate of the intact animal, vital organs of cold-acclimated/adapted fish still require the same amount of energy to fulfill regular energetic demands. Thus, fish must have evolved compensatory mechanisms of metabolic regulation that allow critical tissues to maintain intact functionality in a hypothermic environment.

Previous work showed that exposure to fluctuating temperatures not only results in increased density of mitochondria in tissues, but it also enhances mitochondrial respiration to meet metabolic demands; therefore, ambient temperature fluctuations are likely to result in increased formation of mitochondrial reactive oxygen species (ROS) in ectotherms, including fish [12]. In addition, prolonged exposure to cold may stimulate homeoviscous adaptive responses, such as increased levels of polyunsaturated phospholipids in cellular membranes to maintain membrane fluidity in the brain, which is known to enhance susceptibility to oxidative stress [13]. To combat this increased production and susceptibility to ROS, a protective response has been shown to be initiated in cells of the liver, muscle and central nervous system of fish during cold stress [2,8]. In this response, a suite of small-molecule antioxidants and antioxidative enzymes, including thioredoxin and isoforms of superoxide dismutase (SOD) and glutathione peroxidase (GPx), were found to be upregulated in fish skeletal muscle. However, this response did not prevent accumulation of lipid, protein and DNA oxidation markers, which are slightly elevated in cold-exposed zebrafish muscle [14]. Therefore, oxidative stress represents a major challenge for fish under hypothermic stress, which may also be compounded by the slowdown of cellular repair mechanisms in a cold environment. Thus, in addition to adaptive shifts in metabolism, cell-protective responses are crucial components of the physiological response to hypothermic stress [8].

Resveratrol (3,4′,5-trihydroxy-*trans*-stilbene, RSV) is a phytoalexin that was first isolated from *Veratrum grandiflorum* O. Loes [15]. From studies on mammals, the molecule is known to active sirtuins (SIRTs), which comprise a highly conserved family of NAD-dependent deacetylases [15,16]. Importantly, SIRTs are widely regarded as longevity-related proteins that regulate chromatin epigenetic modifications, metabolism and cellular proliferation [17,18]. Seven SIRT homologues (SIRT1-SIRT7) have been identified in mammals [16,19] and are thought to be centrally involved in metabolic and antioxidative mechanisms, owing to the presence of conserved NAD-binding and catalytic domains [17,19]. According to previous studies, SIRTs mediate signaling through insulin receptor substrate (IRS), peroxisome proliferator-activated receptors (PPARs), forkhead box protein (FOXO) and glyceraldehyde-3-phosphate dehydrogenase (GAPDH) to regulate glucose or lipid metabolism [16,20,21,22]. In addition to regulating metabolism, SIRT1, 2, 3 and 6 are involved in DNA repair and cellular protection against oxidative stress [19]. Molecules, such as SOD, catalase (CAT) and uncoupling protein (UCP), are thought to be involved in these cellular protective machineries [16,19]. Recent studies in fish have revealed that RSV-induced SIRT1 expression might regulate metabolic preference and conversion of biomolecules to cope with ambient stress [23,24]. However, further information regarding RSV-mediated induction of other SIRT homologues and their roles in energy metabolism during cold stress is limited.

The tropical teleost, tilapia, is the second most valuable species in Taiwan aquaculture and one of the most important worldwide; however, with current aquaculture practices, cold winter weather often results in large-scale disease and death of farmed tilapia, causing major economic losses. Since the SIRT protein family is known to participate in metabolic adjustments and cellular protection, we aimed to investigate whether RSV could induce expression of SIRT homologues and SIRT-related genes and stimulate a cold-protective response in tilapia. We characterized the effects of an RSV-diet on known compensatory metabolic and/or cellular protection pathways in tilapia under normal rearing temperatures and hypothermal stress.

## 2. Results

### 2.1. Subsection mRNA Expression of SIRT Homologues in the Liver

**Figure 1:**
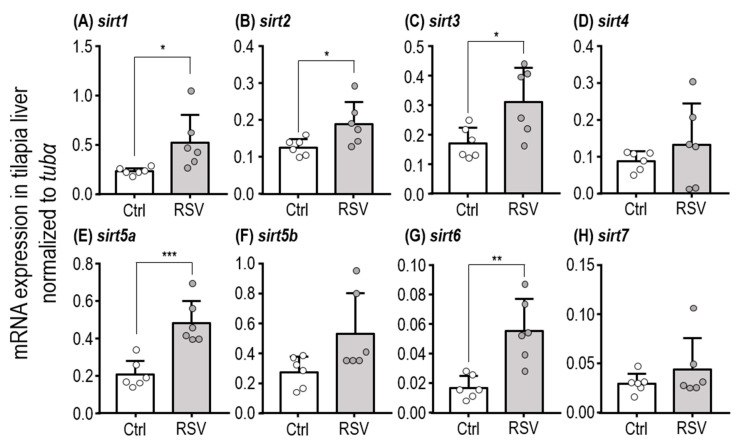
Relative expression levels of SIRT homologues in liver of tilapia fed with the Ctrl and RSV-diet for three days. qPCR analysis was used to compare liver expression levels of *sirt1* (**A**), *sirt2* (**B**), *sirt3* (**C**), *sirt4* (**D**), *sirt5a* (**E**), *sirt5b* (**F**), *sirt6* (**G**), and *sirt7* (**H**) between juvenile tilapia fed with Ctrl and RSV-diets. The tubulin alpha chain (*tubα*) was used as a reference gene. White dots and columns indicate the Ctrl group. Gray dots and columns represent the RSV group. Data are presented as mean ± SD (*n* = 6). Asterisks indicate significant difference (*: *p* < 0.05, **: *p* < 0.01, and ***: *p* < 0.001) between Ctrl and RSV groups.

The mRNA expression levels of SIRT homologues in tilapia liver were evaluated by Quantitative PCR (qPCR). Levels of *sirt6* and *sirt7* transcripts were relatively low compared with other homologues in tilapia liver (Figure S4). Moreover, expression levels of *sirt1*, *sirt2*, *sirt3*, *sirt5a* and *sirt6* in liver were significantly increased in tilapia fed with an RSV-diet for three days compared to those fed with the control diet (Figure 1A–C,E,G; Table 1, Student’s *t*-test; *sirt1*: *p* = 0.0309, *sirt2: p* = 0.0342, *sirt3*: *p* = 0.0213, *sirt5a*: *p* = 0.0007, and *sirt6*: *p* = 0.0022).

### 2.2. SIRT-Target Gene Expression After RSV Induction

**Figure 2:**
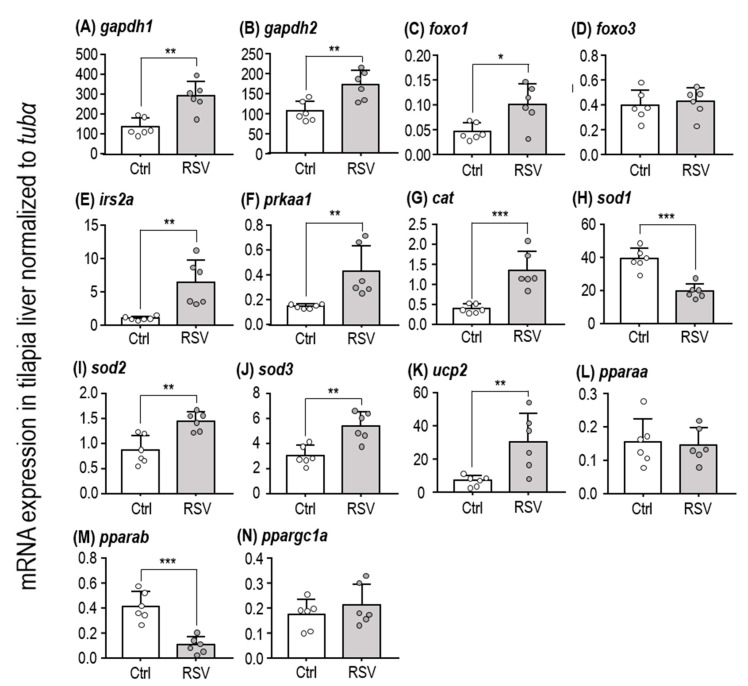
Relative expression levels of SIRT-related homologues in the liver of tilapia fed with the Ctrl and RSV-diet for three days. qPCR analysis was used to compare liver expression levels of *gapdh1* (**A**), *gapdh2* (**B**), *foxo1* (**C**), *foxo3* (**D**), *irs2a* (**E**), *prkaa1* (**F**), *cat* (**G**), *sod1* (**H**), *sod2* (**I**), *sod3* (**J**), *ucp2* (**K**), *pparaa* (**L**), *pparab* (**M**), and *ppargc1a* (**N**) between juvenile tilapia fed with Ctrl and RSV-diets. The tubulin alpha chain (*tubα*) was used as a reference gene. White dots and columns indicate the Ctrl group. Gray dots and columns indicate the RSV group. Data are presented as mean ± SD (*n* = 6). Asterisks indicate significant difference (* *p* < 0.05, ** *p* < 0.01, *** *p* < 0.001) between Ctrl and RSV groups.

SIRTs are known to upregulate expression of several genes. Since we found that an RSV-supplemented diet increases expression of SIRT isoforms, we next measured expression of known SIRT-target genes, including glyceraldehyde-3-phosphate dehydrogenases (*gapdh1* and *gapdh2*) (Figure 2A,B), forkhead box proteins (*foxo1* and *foxo3*) (Figure 2C,D), insulin receptor substrate (*irs2a*) (Figure 2E), AMP-activated protein kinase (*prkaa1*) (Figure 2F), catalase (*cat*) (Figure 2G), superoxide dismutases (*sod1*, *sod2*, and *sod3*) (Figure 2H–J), uncoupling protein 2 (*ucp2*) (Figure 2K), and peroxisome proliferator-activated receptors (*pparaa*, *pparab*, and *ppargc1a*) (Figure 2L–N). After feeding with the RSV-diet for three days, *gapdh1*, *gapdh2*, *prkaa1*, *pparab*, and *foxo1* were significantly upregulated in liver of tilapia (Table 1, Student’s *t*-test; *gapdh1*: *p* = 0.0013, *gapdh2*: *p* = 0.004, *prkaa1*: *p* = 0.0074, *pparab*: *p* = 0.0002, and *foxo1*: *p* = 0.0124). Thus, our protocol for RSV feeding in tilapia appears to trigger the expected protective responses, including those related to energy metabolism and antioxidative enzymes.

### 2.3. Metabolic Response of Intact Tilapia to Cold Conditions

**Figure 3:**
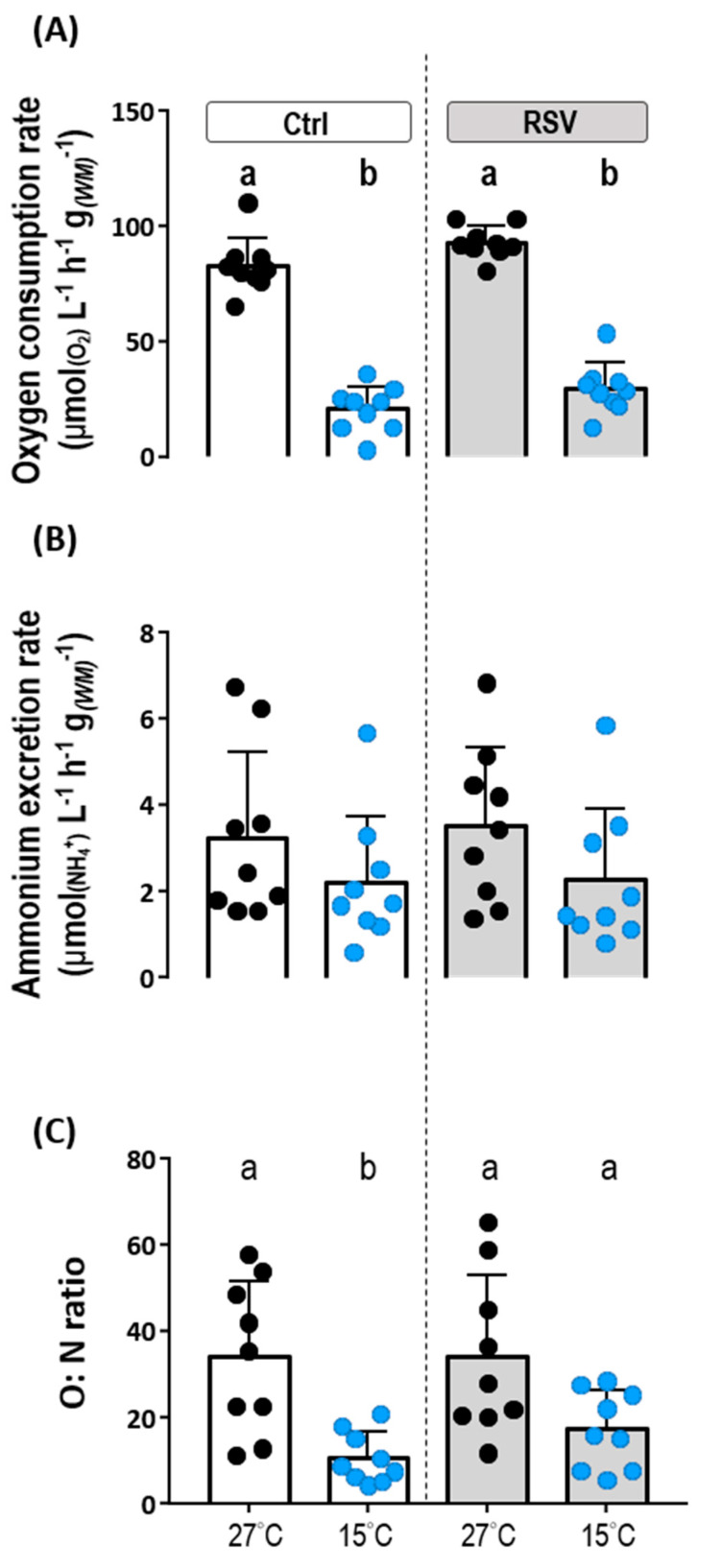
Effects of Ctrl and RSV-diets on oxygen consumption, ammonium extraction rate and O:N ratio at 27 °C and 15 °C. Metabolic rates were measured in fish fed with the Ctrl and RSV-diet by oxygen consumption of intact juvenile tilapia under 27 °C and 15 °C conditions (**A**). NH_4_^+^ excretion rates were determined for fish fed with Ctrl and RSV-diets according to NH_4_^+^ concentrations in bath water of intact juvenile tilapia under 27 °C and 15 °C conditions (**B**). (**C**) O:N ratios were calculated from oxygen consumption rate and NH_4_^+^ excretion rate shown in (**A**,**B**). Black dots and white columns represent the Ctrl-diet group at 27 °C. Blue dots and white columns represent the Ctrl-diet group at 15 °C. Black dots and gray columns represent the RSV-diet group at 27 °C. Blue dots and gray columns represent the RSV-diet group at 15 °C. Data are presented as mean ± SD (*n* = 8 or 9). Different letters indicate significant differences (*p* < 0.05) among different groups (Two-way ANOVA and Tukey’s HSD).

The oxygen consumption rate in intact fish was used as a measure of metabolic rate (RMR) in tilapia. The oxygen consumption rate of control diet Ctrl-fed tilapia at 27 °C was 82.99 ± 12.10 μmol_(O2)_ μmol_(O2)_ L^−1^ h^−1^ g*_(FM)_* ^−1^. Feeding with the RSV-diet for three days at 27 °C did not produce a significant difference in metabolic rate (93.13 ± 7.01 μmol_(O2)_ L^−1^ h^−1^ g*_(FM)_* ^−1^; *p* < 0.0001) (Figure 3A, Table 1). However, both the control and RSV-diet-treated tilapia showed significantly lower oxygen consumption rates after exposure to 15 °C for three days, with the Ctrl-fed fish exhibiting 20.66 ± 9.95 μmol_(O2)_ L^−1^ h^−1^ g*_(FM)_* ^−1^ and the RSV-fed fish showing 29.58 ± 11.12 μmol_(O2)_ L^−1^ h^−1^ g*_(FM)_* ^−1^ (Figure 3A; Ctrl group: *p* < 0.0001, RSV group: *p* < 0.0001 compared to 27 °C). Based on these measurements, exposure to 15 °C lowered oxygen consumption rate by about 68.24% in the RSV-fed fish and 75.11% in their Ctrl-fed counterparts. 

Despite a strong effect of temperature on RMR, no significant differences were found in Ammonium (NH_4_^+^) excretion rates between the animals at 27 °C (3.24 ± 1.99 μmol_(NH4+)_ L^−1^ h^−1^ g*_(FM)_* ^−1^) and 15 °C (2.23 ± 1.51 μmol_(NH4+)_ L^−1^ h^−1^ g*_(FM)_* ^−1^) after three days of exposure (Figure 3B, Table 2; *p* = 0.6061). Furthermore, no significant differences in NH_4_^+^ excretion rates were found between RSV-fed fish at 27 °C (3.54 ± 1.81 μmol_(NH4+)_ L^−1^ h^−1^ g*_(FM)_* ^−1^) and 15 °C (2.27 ± 1.63 μmol_(NH4+)_ L^−1^ h^−1^ g*_(FM)_* ^−1^) (Figure 3B, Table 2; *p* = 0.4215). NH_4_^+^ excretion rates were also not different between control and RSV-fed tilapia at either 27 °C or 15 °C (Figure 3B, Table 2; 27 °C group: *p* = 0.9840, 15 °C group: *p* > 0.9999). 

In addition, the O:N ratio of control and RSV-diet-treated tilapia kept under 27 °C conditions were 34.16 ± 17.58 and 34.22 ± 18.66, respectively (Figure 3C, Table 2). After exposure to 15 °C low temperature conditions for three days, control diet-fed tilapia showed a significantly lower O:N ratio (10.73 ± 5.91) than those at 27 °C (Figure 3C, Table 2; *p* = 0.0059). Interestingly, compared to RSV-diet-fed tilapia kept at 27 °C, the magnitude of the O:N ratio for those maintained at 15 °C was decreased (17.30 ± 9.00), but the difference between the groups was not statistically significant (Figure 3C, Table 2; *p* = 0.7489). Based on these estimations, exposure to low temperature conditions caused the O:N ratio of control diet-fed tilapia to decrease by about 78.69%, while tilapia fed with the RSV-diet exhibited less severe O:N decreases of about 50.45%.

### 2.4. Locomotion under Cold Conditions 

**Figure 4:**
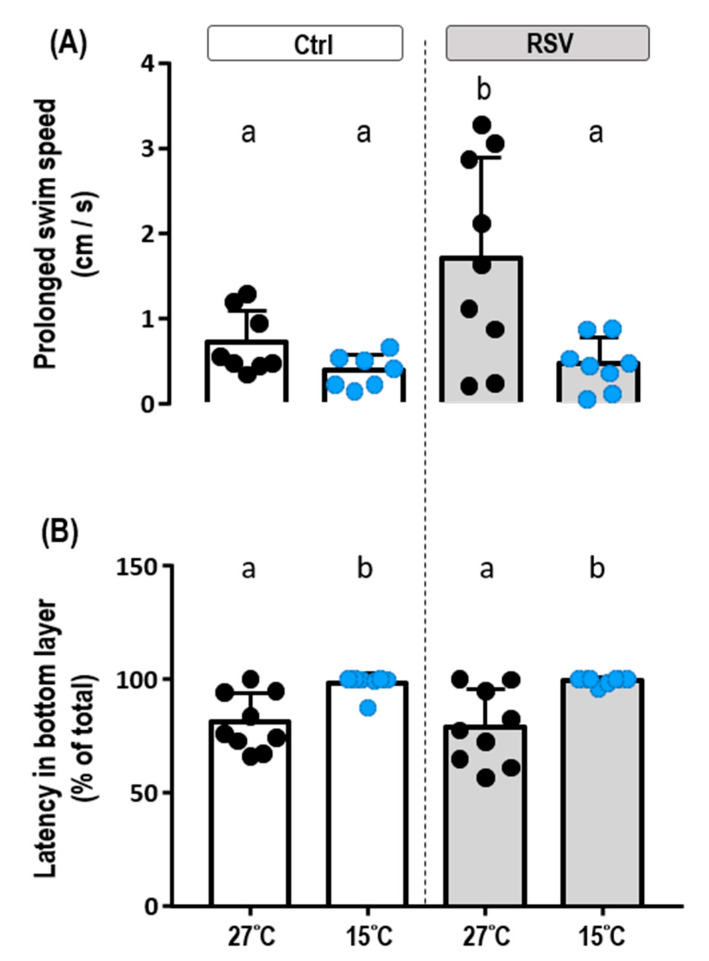
Effects of the Ctrl and RSV-diet on behavior of tilapia at 27 °C and 15 °C. The locomotor activities of fish fed with Ctrl and RSV-diets were estimated by the prolonged swim speed (**A**) and the latency time in the bottom layer (**B**) of juvenile tilapia in a novel tank assay under 27 °C and 15 °C conditions. Black dots and white columns represent the Ctrl-diet group at 27 °C. Blue dots and white columns represent the Ctrl-diet group at 15 °C. Black dots and gray columns represent the RSV-diet group at 27 °C. Blue dots and gray columns represent the RSV-diet group at 15 °C. Data are presented as mean ± SD (*n* = 8 or 9). Different letters indicate significant differences (*p* < 0.05) among different groups (Two-way ANOVA and Tukey’s HSD).

Fish were placed in a novel tank (26 cm long, 8 cm wide, 18 cm deep; water level, 16 cm) (Figure S3A) to assess the locomotion of control- and RSV-fed tilapia at 27 °C and 15 °C. At 27 °C, RSV-fed tilapia showed significantly higher prolonged swim speed (1.70 ± 1.19 cm/s) than the fish fed with control diet (0.71 ± 0.38 cm/s) (Figure 4A, Table 2; *p* = 0.0277). Both control- and RSV-fed fish kept at 15 °C for three days showed significantly lower prolonged swim speeds compared to those kept at 27 °C: control-diet, 0.38 ± 0.19 cm/s; RSV-diet, 0.46 ± 0.31 cm/s (Figure 4A, Table 2; *p* = 0.9962). Based on these measurements, the prolonged swim speed of tilapia fed RSV-diet decreased by 72.94% upon exposure to 15 °C, whereas their control diet-fed counterparts had decreases of 46.47%. A representative trace of locomotor activity from a control-diet tilapia is shown in Figure S3B. In addition, there were no significant differences in upper and bottom layer preference between control- and RSV-fed tilapia at either 27 °C or 15 °C. Notably, however, tilapia did show significantly increased spatial preference for the bottom layer at 15 °C compared to 27 °C; this observation was made for both the control and RSV-diet-fed groups (Figure 4B, Table 2; Ctrl: *p* = 0.0265, RSV: *p* = 0.0043).

## 3. Discussion

The plant polyphenol compound, RSV, is widely considered to be a cellular longevity molecule, and it is known to influence numerous homeostatic processes and stimulate protective mechanisms in several fish models [25,26,27]. Among the physiological machineries induced by RSV, those related to energy metabolism and antioxidative mechanisms are particularly important for stress resistance in most organisms. Whether RSV supplementation is a truly feasible strategy for improving tolerance to ambient stress has not been widely investigated in fish [23]. In the present study, we found that supplementation with a moderate dose of RSV induced expression of several SIRT homologues in tilapia liver, which is a particularly relevant organ since it plays an essential role in energy storage. Higher doses of RSV were found to reduce NH_4_^+^ excretion (Figure S1) and cause irregular and hyperactive behavioral responses in tilapia (data not shown). According to Pereira et. al. (2011), SIRT homologues exhibit tissue specificity in zebrafish, and different dosages and timings for RSV treatment cause diverse effects on expression of SIRT mRNAs in zebrafish brain [28]. In tilapia, expression of all SIRT paralogues could be detected in the liver, and mRNA expression of *sirt1*, *sirt2*, *sirt3*, *sirt5a* and *sirt6* were upregulated by feeding with 25 mg/kg *_WM_* RSV. This information suggests that RSV may be practical for use as an additive in feed.

The role of SIRTs in metabolic homeostasis has been widely studied. Moreover, insulin is an anabolic hormone that acts as a central regulator of the storage and use of metabolic substrates; it is detected by the insulin receptor and signals through IRS adaptor proteins [29]. In addition, FOXO1 has been shown to be a major transcription factor triggering gluconeogenesis [30]. FOXO1 also interacts with peroxisome proliferator activated receptorg-coactivator1-⍺ (PGC1-⍺) to active the transcription of PPARs, which then induce lipid catabolism [31,32]. Therefore, FOXO1 acts as a critical modulator of gluconeogenesis, glycolysis and fatty acid oxidation [29,30]. In mammals, SIRTs function as regulators of energy fuel preference between carbohydrates (e.g., glycogen) and non-carbohydrate carbon substrates (e.g., lipids) under stress [19,33]. For example, SIRT1 promotes FOXO1 deacetylation and PPARs activation to stimulate gluconeogenesis and fatty acid oxidation, thus inhibiting lipogenesis in organisms under hypoglycemia stress [19,20]. SIRT1 could further contribute to insulin signaling by upregulating IRS2 deacetylation in mammalian pancreatic β cells [33,34]. Additionally, SIRT2 enhances lipolysis to support gluconeogenesis via FOXO1 deacetylation in adipose tissue [19]. SIRT5 induces gluconeogenesis and glycolysis via GAPDH demalonylation [33], while SIRT6 plays a role in glycolysis inhibition and lipid catabolism similar to its activator, SIRT1 [20]. Together, these studies show that each SIRT family member modulates metabolic processes via different but overlapping mechanisms. In this study, increased expression levels of *foxo1*, *pparab*, *irs2a*, *gapdh1*, and *gapdh2* suggest that RSV modulates the metabolic trade-off between glucose and lipid metabolism via activated expression of *sirt1*, *sirt2*, *sirt3* and *sirt6*. These results infer that the moderate dosage of RSV supplementation used in the present study might affect the insulin and FOXO signaling pathway to alter gluconeogenesis, glycolysis and lipolysis.

In addition to insulin, protein kinase AMP-activated/5′-AMP-activated protein kinase (PRKA/AMPK) acts as another central cellular energy sensor and a master energy homeostasis regulator. As such, AMPK is known to affect gluconeogenesis, glycolysis and fatty acid oxidation, as well as fatty acid and protein synthesis [22]. Notably, AMPK has been shown to increase insulin secretion in the pancreatic β cells of mice [29]. However, the reason AMPK induces both FOXO1 and insulin to stimulate seemingly opposed metabolic process is still unclear. SIRT1 and AMPK both control many common cell signaling molecules, such as PPARs and FOXO1, which affect not only energy homeostasis but also oxidative stress responses [35,36,37]. A feedback adaptive loop has been proposed to exist between SIRT1 and AMPK [22,36,38]. In addition, a previous study showed that different doses of RSV could drive diverse effects of this SIRT1-AMPK loop [39]. In the present study, we found parallel increases in expression of *prkaa* and *sirt1*; thus, detailed mechanistic studies on the presence and function of a SIRT1-AMPK loop in tilapia organs may be warranted.

An accumulation of ROS with coincident enhancement of metabolic demand was previously reported in ectothermic teleost zebrafish under ambient hypothermic stress [8]. Furthermore, hypothermia-induced cellular oxidative stress is known to cause lipid and protein oxidation and 8-oxodG-DNA damage [40,41]. To limit such damage, a protective signaling pathway is induced, resulting in increased levels of a suite of small-molecule antioxidants and antioxidative enzymes [40]. PPARs are ligand-activated transcription factors of the nuclear receptor superfamily, which are known to regulate the expression of target genes involved in lipid and energy metabolism [42]. PPARs also activate the expression of antioxidant enzymes/protein, including SOD, CAT and UCP, to mitigate increased ROS [8,13]. In the present study, we found that several of these antioxidants, i.e., *cat*, *ucp2*, *sod1*, *sod2*, and *sod3,* were increased by RSV feeding. A previous study in zebrafish also revealed that promotion of glycolysis during cold exposure is beneficial for physiological maintenance [8]. Increased glucose utilization and glycolysis rates may help compensate for the hypothermia-induced oxidation stress. Such adaptive metabolic shifts and cell-protective mechanisms in liver are crucial for physiological homeostasis under hypothermic stress.

Since the cellular energy reallocation is necessary to successfully cope with hypothermic conditions, oxygen consumption and swimming performance are both susceptible to change with temperature in ectothermic fish [43,44]. For example, most fish exhibit decreased locomotor activity under cold stress as an energy conservation measure [43,45]. The term ‘metabolism’ is often used to indicate the sum of all biochemical catabolic and anabolic processes in an organism. Oxygen consumption rate of intact fish in the rest condition is regarded as a suitable parameter to reflect metabolic rate, which indirectly indicates overall energy demand. Additionally, catabolism of amino acids is a major energy source in fishes, since most are ammonotelic [46,47]. Therefore, changes in O:N ratio provide a reliable indicator of corresponding changes in the utilization of different energy substrates [48]. Tilapia is a common tropical freshwater fish, and the lowest temperature at which they can survive is around 15 °C [49,50]. Although both Ctrl-fed and RSV-fed tilapia showed decreased metabolism, according to oxygen consumption rate when kept at 15 °C for three days, a decreased O:N ratio was observed in control tilapia at 15 °C, but the O:N ratio was not significantly lower in RSV-fed counterparts at 15 °C compared to 27 °C. This observation implies that RSV supplementation may modify energy substrate utilization under hypothermic conditions. Furthermore, none of the fish on RSV supplementation swam erratically at 15 °C, in contrast to their control diet-fed counterparts (Table S2). Therefore, RSV supplementation appears to activate SIRTs as well as cellular protection machineries to allow ectothermic tilapia to overcome ambient hypothermic challenge without obviously altered behavioral phenotypes. 

RSV-related research is not extensive in fish. Only a few studies have examined RSV enhancement of glucose tolerance in fish [24,51]. In aquaculture, metabolism and antioxidative molecules are both important targets that may improve cold tolerance in fish. The present study clearly shows that RSV supplementation can influence these adaptive features to promote the maintenance of physiological homeostasis in cold-exposed fish. Based on the lack of changes in ammonium excretion and behavioral phenotypes after RSV-diet feeding, we expect that supplementing feed with RSV will not result in significant negative effects on regular fish culture. In subsequent studies, the detailed mechanistic linkage between RSV-induced SIRT homologues, related metabolic/antioxidative molecules, and intact metabolites composition should be carefully characterized to better understand the mechanisms of RSV action on aquatic animals.

## 4. Materials and Methods

### 4.1. Animal Breeding and Experimental Design

Juvenile tilapia (*Oreochromis mossambicus*; body length: 6.8 ± 0.6 cm; wet weight: 4.4 ± 1.1 g) were used to avoid the influence of sex in the study. *Oreochromis mossambicus* were obtained from the Marine Research Station, Institute of Cellular and Organismic Biology of Academia Sinica. Thirty fish were kept in a transparent tank (90 cm long, 34 cm wide and 49 cm deep; water level, 45 cm) with a fresh-water circulating system. The fish were hold at 27 ± 1 °C under a 12L:12D photoperiod. Fish were fed with artificial feed pellets (Fu-So, Taipei, Taiwan) or RSV-feed pellets once per day. RSV was purchased from Sigma-Aldrich (St Louis, MO, USA). According to a pilot test (Figure S1) and previous studies in tilapia [23], 25 mg/kg *_WM_* dietary supplementation of RSV was selected as the experimental dosage. All fish were fasted for 24 h before experiments. The experimental protocols were approved by the Academia Sinica Institutional Animal Care and Biosafety Committee (approval no. BSF0418-00003847, approved on 12 March 2018).

The effects of RSV on expression of SIRT homologues and SIRT-related genes in tilapia liver were evaluated according to the experimental design shown in Figure S2A. Liver was collected from control fish and stored at −80 °C for total RNA extraction and real-time quantitative PCR analysis (qPCR) on the first day before feeding with the RSV-diet. MS-222 (Ethyl 3-aminobenzoate methanesulfonate salt, 0.03%) was used for euthanasia. Afterward, tilapia were fed with control or RSV-diet for three days. Tilapia fed with the control or RSV-diet (three days treatment) were then directly transferred to 15 °C or kept at 27 °C for another three days. Because fish generally eat less in cold temperatures, tilapia were not fed during the three days after transfer to 15 °C and 27 °C (Figure S2B). Oxygen consumption rate, NH_4_^+^ excretion rate and swimming activity were measured at the end of the three days without feed.

### 4.2. Purification of mRNA

The total RNA was extracted from fish liver with Trizol Reagent (Invitrogen, Carlsbad, CA, USA). Genomic DNA contamination was removed with DNase I (Promega, Madison, WI, USA). The mRNA for real-time quantitative PCR (qPCR) was purified with QuickPrep Micro mRNA Purification kit (Amersham Pharmacia, Piscataway, NJ, USA). The quality of mRNA was determined at 260 and 280 nm with a spectrophotometer (Hitachi U-2000, Tokyo, Japan). The RNA integrity was further checked with an Agilent 2100 Bioanalyzer (Agilent Technologies, Santa Clara, CA, USA). All RNA samples were stored at −20 °C prior to use.

### 4.3. Real-Time Quantitative PCR (qPCR)

The mRNA was reverse-transcribed with Superscript reverse transcriptase IV (Invitrogen, Carlsbad, CA, USA) for cDNA synthesis. The mRNA expression levels for target genes were measured using a Roche LightCycler H480 System (Roche Applied Science, Mannheim, Germany). Primers for all genes were designed with PrimerQuest Tool (Integrated DNA Technologies, USA) and are shown in Table S1. cDNA (3.2 ng), 50 nM primer, and LightCycler^®^ 480 SYBR Green I Master (Roche) in a final volume of 10 μL were added to each PCR reaction. All the PCR reactions were performed as follows: one cycle of 50 °C for 2 min, 95 °C for 10 min, 40 cycles of 95 °C for 15 s and 60 °C for 1 min. Control reactions were conducted with sterile water to replace cDNA sample as non-template control (NTC). The standard curve of each gene was confirmed to be in a linear range with tubulin alpha chain (*tubα*) as a reference gene.

### 4.4. Oxygen Consumption and Ammonium Excretion

Oxygen consumption rates were measured approximately 24 h after the previous feeding and followed procedures modified from [52,53]. Each test subject was gently placed in a 3-L glass respiration chamber containing ultraviolet, light-sterilized, fully aerated fresh water. The respiration chambers were equipped with fiber-optic oxygen sensors (Oxy-4mini, PreSens GmbH, Regensburg, Germany) and operated while closed and housed in opaque containers to reduce ambient influences. Oxygen concentration was monitored every 5 s for 1.5 h after probes were calibrated with oxygen saturated fresh water and water with zero oxygen (10% sodium bisulphate solution). Oxygen concentration decreased linearly over time. This decrease was used to calculate oxygen consumption in units of µmol_(O2)_ L^−1^ h^−1^ g*_WM_*^−1^. One chamber without fish was used to measure background respiration from bacteria or other microorganisms.

Ammonium excretion rates were determined from NH_4_^+^ concentration measurements made prior to and following the oxygen consumption measurements. Before and after closing the swim tunnel chambers, a 1-mL water sample was removed, and 250 μL of working reagent (21 mM sodium tetraborate, 0.063 mM sodium sulfite, and 50 mL/L orthophthaldialdehyde in ethanol) was added. Testing samples were then incubated for 2 h at room temperature in the dark until fluorescence was determined at an excitation wavelength of 360 nm and emission wavelength of 422 nm (Spectramax multi-mode microplate readers i3, Molecular Device, USA. Additionally, a separate glass chamber was incubated without tilapia to determine background readings of filtered fresh water. Ammonium excretion rates were calculated as μmol_(NH4_^+^_)_ L^−1^ h^−1^ g*_WM_*^−1^.

The atomic ratio of oxygen uptake to excreted nitrogen was calculated from respiration and ammonium excretion rates:

O:N = 2MO_2_ (NH_4_^+^ excretion)^−1^

### 4.5. Locomotion

Individual tilapia were transferred from their home tank with careful handing to the experimental tank for 15 min habituation. A novel tank (26 cm long, 8 cm wide and 18 cm deep; water level, 16 cm; Figure S3A) with a white plastic floor was used to evaluate the locomotion activity of tilapia swimming. According to our pilot test, the representing swimming features and responsive behavior in the experimental novel tank are similar to that in the home tank. The total swimming distance in 5 min after habituation was calculated from a recording made with a video camera (UI-3240CP Rev.2, Ids, Germany). The prolonged swim speed and spatial preference were calculated. Movement was tracked with Ethovision XT motion tracking software (v. 7.0, Noldus, The Netherlands), as shown in Figure S3B.

### 4.6. Statistical Analysis

GraphPad Prism 7.00 (GraphPad, San Diego, CA, USA) was used for statistical analyses. Values are presented as mean ± SD. Student’s *t*-test was used to analyze the difference between mRNA gene expression levels with and without RSV-diet feeding. Two-way ANOVA and Tukey’s HSD test were used to determine the significance of effects of different temperatures and diets on oxygen consumption rate, ammonia extraction rate, and locomotion activity of fish.

## 5. Conclusions

The tropical teleost, tilapia, is of high trophic and commercial importance in current aquaculture practices; however, cold winter weather often results in large-scale disease and death of farmed tilapia. A naturally plant phytoalexin compound, resveratrol (RSV), that is inferred to stimulate complex cellular activities, was proved to serve as a feasible diet supplement for fish in this study, and may benefit tropical tilapia to survive in temperatures at the lower limit of thermal tolerance. Feeding tilapia with RSV-supplemented diet apparently promoted cellular longevity molecule—sirtuins expressions in liver tissue. In addition, our work also represents an important step towards a better understanding regarding RSV supplement allowing tropical tilapia to perform „adaptive metabolic shift” that shown in those molecular responses, and therefore adjust respective swimming activity under cold stress. Consequently, RSV-induced sirtuins expressions in tilapia liver may benefit to improve aquaculture practice under winter cold front stress.

## Figures and Tables

**Table 1:** Statistical comparisons (Student’s *t*-test) of target gene expression in livers of tilapia fed with Ctrl and RSV-diets.

Gene	*t*	df	*p-*Value
*sirt1*	2.509	10	0.0309 *
*sirt2*	2.452	10	0.0342 *
*sirt3*	2.727	10	0.0213 *
*sirt4*	0.9317	10	0.3735
*sirt5a*	4.808	10	0.0007 ***
*sirt5b*	2.196	10	0.0528
*sirt6*	4.071	10	0.0022 **
*sirt7*	1.052	10	0.3176
*gapdh1*	4.416	10	0.0013 **
*gapdh2*	3.71	10	0.004 **
*foxo1*	3.041	10	0.0124 *
*foxo3*	0.448	10	0.6637
*irs2a*	3.843	10	0.0033 **
*prkaa1*	3.345	10	0.0074 **
*cat*	4.934	10	0.0006 ***
*sod1*	6.1	10	0.0001 ***
*sod2*	4.099	10	0.0021 **
*sod3*	4.195	10	0.0018 **
*ucp2*	3.282	10	0.0083 **
*pparaa*	0.2313	10	0.8217
*pparab*	5.691	10	0.0002 ***
*ppargc1a*	0.9309	10	0.3739

Asterisks indicate significant difference (*: *p* < 0.05, **: *p* < 0.01, and ***: *p* < 0.001) between Ctrl and RSV groups.

**Table 2:** Statistical comparisons (two-way ANOVA) of metabolic and behavioral parameters from tilapia fed with Ctrl or RSV-diets and maintained at 27 °C or 15 °C.

	df	MS	*F*	*p*
**Oxygen consumption rate**				
Feeding	1	816	7.811	0.0087
Temperature	1	35651	340.9	<0.0001
Feeding × Temperature	1	3.317	0.0317	0.8598
Error	32	104.6		
**Ammonium excretion rate**				
Feeding	1	0.2527	0.0831	0.7749
Temperature	1	11.83	3.89	0.0573
Feeding × Temperature	1	0.1439	0.0473	0.8291
Error	32	3.04		
**O: N ratio**				
Feeding	1	99.18	0.5132	0.4789
Temperature	1	3664	18.96	0.0001
Feeding × Temperature	1	95.17	0.4925	0.4879
Error	32	193.2		
**Prolong swim speed**				
Feeding	1	2.296	4.873	0.0356
Temperature	1	4.916	10.43	0.0032
Feeding × Temperature	1	1.678	3.561	0.0696
Error	28	0.4712		
**Latency in bottom layer**				
Feeding	1	1.911	0.0151	0.9028
Temperature	1	2856	22.67	<0.0001
Feeding × Temperature	1	21.97	0.1744	0.6793
Error	29	126		

## Supplementary Materials

Supplementary materials can be found at https://www.mdpi.com/1422-0067/21/9/3338/s1. Figure S1. The effect of different RSV dosages on juvenile tilapia oxygen consumption (A) and ammonium excretion (B) rate. Values that are significantly different (*p* < 0.05) among groups are indicated by different letters. Data are presented as mean ± SD (*n* = 5–9). Figure S2. Experimental design. Tilapia were reared at 27 °C and fed with control diet before experiments. (A) The effects of RSV-diet on gene expression of SIRT homologues and SIRT-related genes in tilapia liver were estimated after three days of RSV-diet feeding and compared with the fish fed on control diet. (B) After feeding with the Ctrl and RSV-diets for three days, two treatment groups were subdivided into 27 °C and 15 °C conditions and fasted for three days. Physical and physiological features were assessed after the temperature treatments. Figure S3. Behavioral experiment set-up. (A) An individual tilapia was placed in a novel tank (26 cm long, 8 cm wide and 18 cm deep; water level: 16 cm) and swimming performance was recorded. (B) Trajectory tracking was performed for 5 min, and tracks were used to analyze the prolonged swim speed and spatial preference of fish. Figure S4. Sirtuin homologue expression in tilapia liver. qPCR analysis of relative mRNA expression levels of sirtuin homologues in liver of tilapia juveniles. Data are expressed as mean ± SD (*n* = 6). Table S1. qRT-PCR primer sequences. Table S2. Swimming phenotypes of Ctrl- and RSV-fed tilapia at 15 °C for three days.

## Abbreviations

RSV	Resveratrol
ROS	Reactive oxygen species
SOD	superoxide dismutase
GPx	glutathione peroxidase
SIRTs	sirtuins
IRS	insulin receptor substrate
PPARs	peroxisome proliferator-activated receptors
FOXO	forkhead box protein
GAPDH	glyceraldehyde-3-phosphate dehydrogenase
CAT	catalase
UCP	uncoupling protein
