# Supplementary material for: Resveratrol Induces Expression of Metabolic and Antioxidant Machinery and Protects Tilapia under Cold Stress
Source: Int J Mol Sci. 2020 May 8;21(9):3338. doi: 10.3390/ijms21093338 (PMC7246431; doi:10.3390/ijms21093338)
Supplement: Supplementary file 1 [file ijms-21-03338-s001.pdf]

Supplemental information to:

Resveratrol Induces Expression of Metabolic and Antioxidant Machinery and Protects Tilapia under Cold Stress

Min-Chen Wang [1,2,†], Yu-Chun Wang [3,†], Hui-Wen Peng [1], Jinn-Rong Hseu [3], Guan-Chung Wu [4], Ching-Fong Chang [4] and Yung-Che Tseng [1,*]

[1] Marine Research Station, Institute of Cellular and Organism Biology, Academia Sinica, 262, Taiwan; mcwinlab@gmail.com (M.-C.W.) b5pickfa@gmail.com (H.-W.P.)

[2] Taiwan International Graduate Program, National Taiwan Normal University, 116, Taiwan

[3] Planning and Information Division, Fisheries Research Institute, COA, 202, Taiwan; ycwang@mail.tfrin.gov.tw (Y.-C.W.); jrhseu@mail.tfrin.gov.tw (J.-R.H.)

[4] Department of Aquaculture, National Taiwan Ocean University, 202, Taiwan;

gcwu@mail.ntou.edu.tw (G.-C.W.); b0044@email.ntou.edu.tw (C.-F.C.)

* Correspondence: yctseng@gate.sinica.edu.tw

† These authors contributed equally to this work

*To whom correspondence should be addressed:

E-Mail: yctseng@gate.sinica.edu.tw

Tel: +886-3-9880544#14

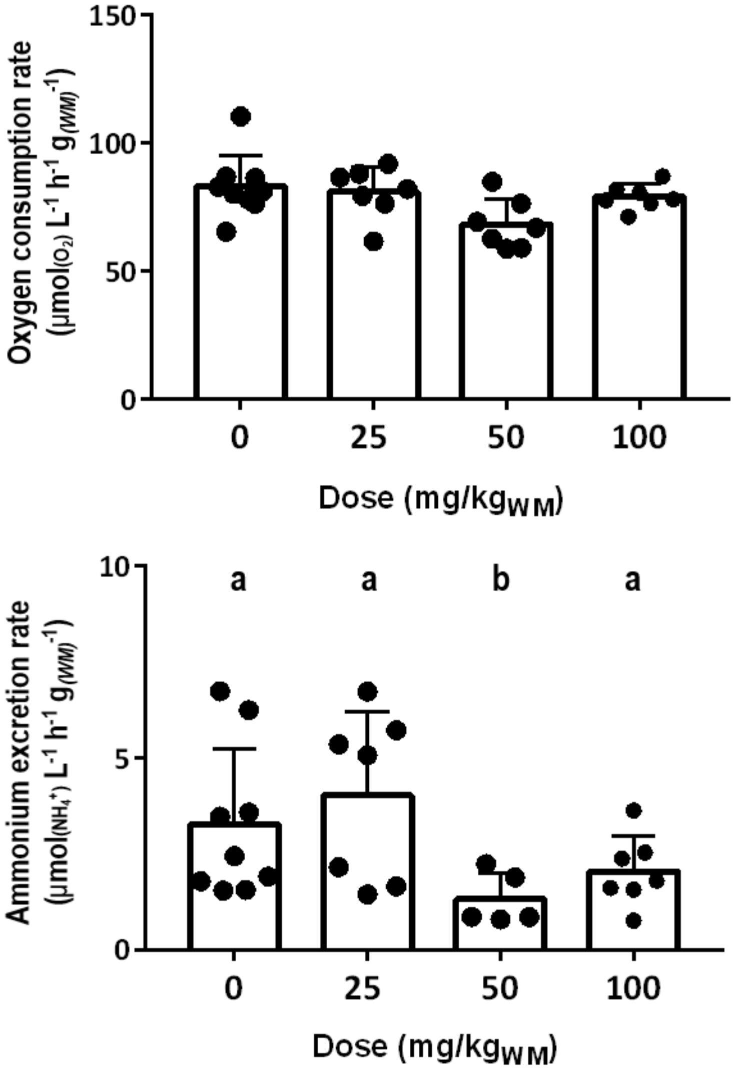

Figure S1. The effect of different RSV dosages on juvenile tilapia oxygen consumption (**A**) and ammonium excretion (**B**) rate. Values that are significantly different ($p < 0.05$) among groups are indicated by different letters. Data are presented as mean ± SD (n = 5–9).

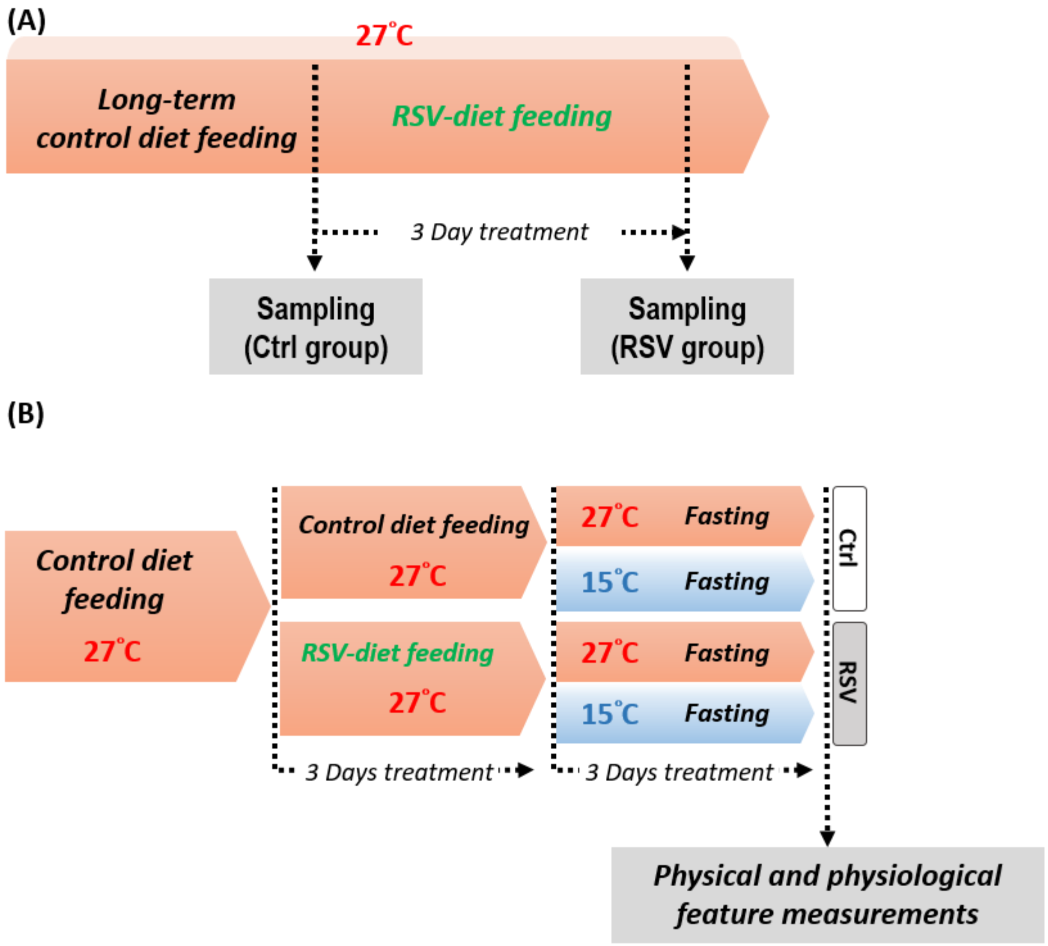

Figure S2. Experimental design. Tilapia were reared at 27 °C and fed with Ctrl-diet before experiments. (**A**) The effects of RSV-diet on gene expression of SIRT homologues and SIRT-related genes in tilapia liver were estimated after three days of RSV-diet feeding and compared with the fish fed on control diet. (**B**) After feeding with the Ctrl and RSV-diets for three days, two treatment groups were subdivided into 27 °C and 15 °C conditions and fasted for three days. Physical and physiological features were assessed after the temperature treatments.

(A)

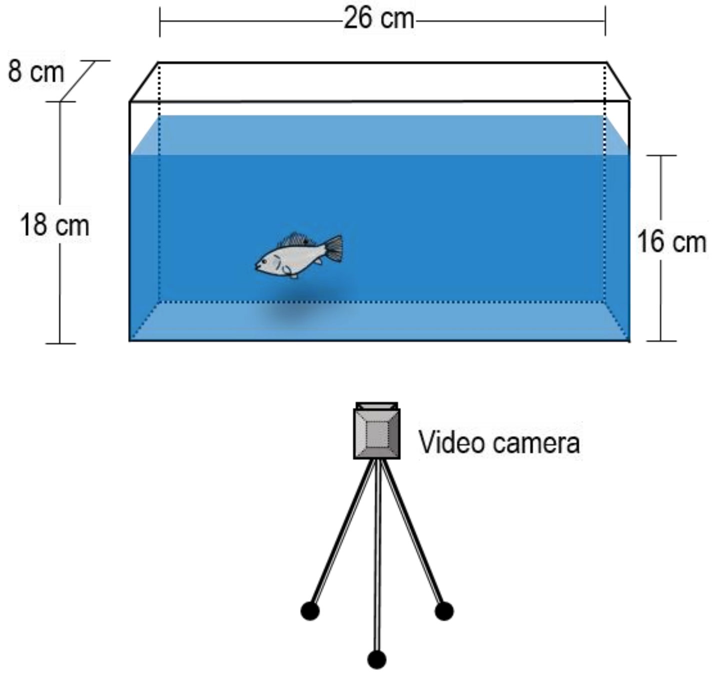

(B)

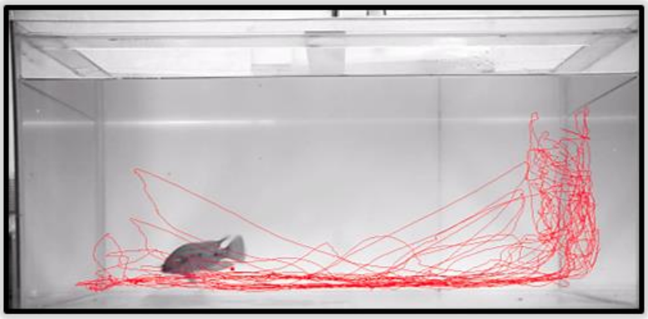

Figure S3. Behavioral experiment set-up. (**A**) An individual tilapia was placed in a novel tank (26 cm long, 8 cm wide and 18 cm deep; water level: 16 cm) and swimming performance was recorded. (**B**) Trajectory tracking was performed for 5 min, and tracks were used to analyze the prolonged swim speed and spatial preference of fish.

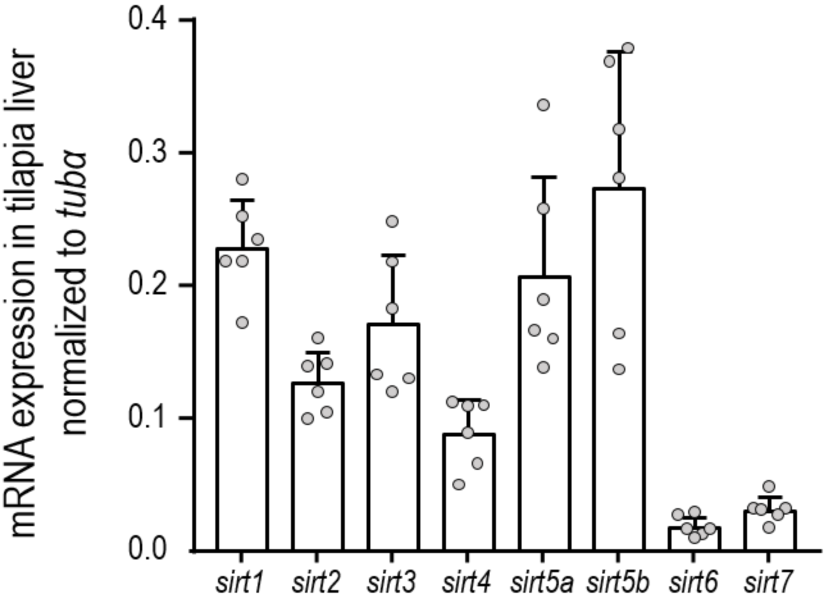

Figure S4. Sirtuin homologue expression in tilapia liver. qPCR analysis of relative mRNA expression levels of sirtuin homologues in liver of tilapia juveniles. Data are expressed as mean ± SD (n = 6)

Table S1. qRT-PCR primer sequences.

Gene Name	Abbreviation		Primer Sequence (5′→3′)	Primer Efficiency (%)	Amplicon Size (bp)	Accession Numbers
Sirtuin-1	SIRT1	F	AACTTGACGACACCGCTGTCTTTG	99	137	XM_005473846
		R	GCTTGCATGTGAGGACCTGTCATC			
Sirtuin-2	SIRT2	F	CATGGAGAAAGCAGGGCAGGTTAATC	93	161	XM_003449264
		R	GCTCTTCCTTCCATCCCAGAAGATCA			
Sirtuin-3	SIRT3	F	AGTTCTGTCCAGACATGCACGATCT	98	124	XM_005457371
		R	CCTGTTGAACTGTTGCTCCCGTATG			
Sirtuin-4	SIRT4	F	TTCAGAGTTCCCTCCTGTGATGACT	96	154	XM_025907266
		R	ACACCTGTAAAGATGACCCCACAAC			
Sirtuin-5a	SIRT5a	F	CCACCGAGCTGGATCCAAAAAC	94	179	XM_003457306
		R	TGGGCAAATTCTGGACTGGGAT			
Sirtuin-5b	SIRT5b	F	CGGCTCTGTGCTCTGATAGAAGGT	95	190	XM_003438119
		R	GACTCATGTTGTGCCAAAGCTTGAG			
Sirtuin-6	SIRT6	F	CGCAAGGGTGGCAAACTGGTTAT	97	197	XM_003437978
		R	TTGACATCAGCGGTGGTCTCAGT			
Sirtuin-7	SIRT7	F	GCATGAACAGACCAGCAAGCAAAA	93	100	XM_003455810
		R	CACTTGCCGTGAATTTTCAGCACA			
Glyceraldehyde 3-phosphate dehydrogenase 1	GAPDH1	F	TCATCCCTGAGCTCAATGGCAA	98	187	XM_005455438
		R	AGACCTGGTGCTCTGTGTATCC			
Glyceraldehyde 3-phosphate dehydrogenase 2	GAPDH2	F	TCTGGGATACACAGAGGACCAGGT	98	102	XM_003452690
		R	AAGTTGTCGTTGAGTGCAATGCCA			
Protein kinase AMP-activated catalytic subunit alpha 1	PRKAA1	F	GTGCAGCGATAGTCAAGCCTCAC	96	127	NM_001319868
		R	CTCCTAACACCCTGGTGCTTGGA			
Peroxisome proliferator-activated receptor alpha	PPARA	F	CATGATGGAGCCCAAATTCCAGTTTG	91	153	NM_001290066
		R	ATGCTTTCCTGCAACTGCTCTACTAG			
Peroxisome proliferator activated receptor alpha	PPARAB	F	TCCCATCACCACAATGGTCGACA	94	175	XM_003443920
		R	TGGGACACCAAAGGAGCTGAGAG			

PPARG related coactivator 1	PPARGC1a	F<br>R	TACCTAACCGCCACCGATGACATT<br>CATGCCACTCTTCTCCCTGCTACT	92	96	XM_005468189
Forkhead box protein 1	FOXO1	F<br>R	CAAACGCCGAGTTCATCAGAAACCT<br>GGGTAATGATGCACACAGTTGCTGT	92	121	XM_025910765
Forkhead box protein 3	FOXO3	F<br>R	ACAACCACAATCACAGCTCTCTGAG<br>CGATCCTGATAGTTCCCTCCATTGC	96	196	XM_005454618
Insulin receptor substrate 2a	IRS2A	F<br>R	TGGGAGGTCATCTCTAGCAGACTACA<br>CCCTGGGAGTCTTTCTCACTATCCAC	90	168	XM_003440900
Catalase	CAT	F<br>R	GCGACAGAGACTTTGCCAGAAC<br>ACGGCTGTAAACATGCAAGGTG	90	181	XM_019361816
Mitochondrial uncoupling protein 2	UCP2	F<br>R	TTCGTTACCACAGTGATCGCCT<br>ACGAGGGCACGAATCCTTTGTA	91	154	XM_003452255
Superoxide dismutase 1	SOD1	F<br>R	CACCCTCACAGGTCCTGACTCC<br>AATGACTCCACAGGCCAGACGT	92	136	XM_003446807
Superoxide dismutase 2	SOD2	F<br>R	GAACATGCTTTGCAGAGCTGGACA<br>CAGCTGCATGATCTCTGCACTGAC	95	160	XM_003449940
Superoxide dismutase 3	SOD3	F<br>R	GAAAGTCAAGGTCCTCCTCCGGTT<br>TCTTCCCTTGCTGAGGCTCAAAGT	98	194	XM_003454189
Tubulin alpha chain	TUBα	F<br>R	GCCTTCAGCAACCGATTCTT<br>CAGCATGCATTGCCCATTTG	98	115	XM_019352023

F, forward primer; R, reverse primer

Table S2. Swimming phenotypes of Ctrl- and RSV-fed tilapia at 15 °C for three days.

Phenotypes	Ctrl Diet Feeding	RSV-Containing Diet Feeding
Erratic Swimming	(6/9)	(0/9)

Numbers in parentheses indicate (number of fish with phenotype/total experimental fish)
